# Supplementary material for: Crawling and Gliding: A Computational Model for Shape-Driven Cell Migration
Source: PLoS Comput Biol. 2015 Oct 21;11(10):e1004280. doi: 10.1371/journal.pcbi.1004280 (PMC4619082; doi:10.1371/journal.pcbi.1004280)
Supplement: S1 Code — (ZIP) [file pcbi.1004280.s012.zip › release/tst/doc/html/functions_vars.html]

Tissue Simulation Toolkit: Class Members - Variables


Tissue Simulation Toolkit
 0.1.4.1


- Main Page
- Namespaces
- Classes
- Files

- Class List
- Class Hierarchy
- Class Members

- All
- Functions
- Variables
- Related Functions

- a
- b
- c
- d
- e
- g
- j
- l
- m
- n
- o
- p
- r
- s
- t
- v
- x
- y

### - a -

- aa1
  : Dir
- aa2
  : Dir
- alive
  : Cell
- alt\_sigma
  : PDE
- amount
  : Cell
- area
  : Cell

### - b -

- bb1
  : Dir
- bb2
  : Dir
- border\_energy
  : Parameter

### - c -

- capacity
  : Cell
- cell
  : Dish
- chem
  : Cell
- chemotaxis
  : Parameter
- colour
  : Cell
- colour\_of\_birth
  : Cell
- conn\_diss
  : Parameter
- CPM
  : Dish

### - d -

- datadir
  : Parameter
- date\_of\_birth
  : Cell
- daughter
  : Cell
- decay\_rate
  : Parameter
- diff\_coeff
  : Parameter
- divisions
  : Parameter
- dt
  : Parameter
- dx
  : Parameter

### - e -

- extensiononly
  : Parameter

### - g -

- grad
  : Cell
- graphics
  : Parameter
- growth\_threshold
  : Cell

### - j -

- J
  : Cell
- Jtable
  : Parameter

### - l -

- lambda
  : Parameter
- lambda2
  : Parameter
- layers
  : PDE
- lb1
  : Dir
- lb2
  : Dir
- length
  : Cell

### - m -

- maxsigma
  : Cell
- maxtau
  : Cell
- mcs
  : Parameter
- mother
  : Cell

### - n -

- n\_chem
  : Parameter
- n\_copies
  : Cell
- n\_init\_cells
  : Parameter
- neighbours
  : Parameter

### - o -

- owner
  : Cell

### - p -

- pde\_its
  : Parameter
- PDEfield
  : Dish
- periodic\_boundaries
  : Parameter
- polarvec
  : Cell

### - r -

- relaxation
  : Parameter
- rseed
  : Parameter

### - s -

- saturation
  : Parameter
- secr\_rate
  : Parameter
- sigma
  : Cell
  , CellularPotts
  , PDE
- size\_init\_cells
  : Parameter
- sizex
  : CellularPotts
  , Parameter
  , PDE
- sizey
  : CellularPotts
  , Parameter
  , PDE
- spins\_converted
  : CellularPotts
- storage\_stride
  : Parameter
- store
  : Parameter
- subfield
  : Parameter
- sum\_x
  : Cell
- sum\_xx
  : Cell
- sum\_xy
  : Cell
- sum\_y
  : Cell
- sum\_yy
  : Cell

### - t -

- T
  : Parameter
- target\_area
  : Cell
  , Parameter
- target\_length
  : Cell
  , Parameter
- tau
  : Cell
- times\_divided
  : Cell

### - v -

- v
  : Cell
- vecadherinknockout
  : Parameter

### - x -

- x
  : co
  , Point
- x1
  : li
- x2
  : li

### - y -

- y
  : co
  , Point
- y1
  : li
- y2
  : li


---

Generated on Thu Aug 14 2014 22:04:02 for Tissue Simulation Toolkit by  

 1.8.6
